# Clinical characteristics and patterns of healthcare utilization in patients with painful neuropathic disorders in UK general practice: a retrospective cohort study

**DOI:** 10.1186/1471-2377-12-8

**Published:** 2012-03-06

**Authors:** Ariel Berger, Alesia Sadosky, Ellen Dukes, John Edelsberg, Gerry Oster

**Affiliations:** 1 Policy Analysis Inc. (PAI), Four Davis Court Brookline, MA 02445, USA; 2 Pfizer Inc, 235 East 42nd Street, New York, NY 10017, USA

**Keywords:** Neuralgia, Nerve pain, Peripheral neuropathies, Pharmacotherapy, Analgesia, Health services research

## Abstract

**Background:**

Clinical characteristics and patterns of healthcare utilization in patients with painful neuropathic disorders (PNDs) who are under the care of general practitioners (GPs) in the UK are not well understood.

**Methods:**

Using a large electronic UK database, we identified all adults (age ≥ 18 years) with any GP encounters between 1 January 2006 - 31 December 2006 at which a diagnosis of PND was noted ("PND patients"). An age-and gender-matched comparison group also was constituted consisting of randomly selected patients with one or more GP encounters-but no mention of PNDs-during this period. Characteristics and patterns of healthcare utilization of patients in the two groups were then examined over the one-year study period.

**Results:**

The study sample consisted of 31,688 patients with mention of PNDs and an equal number of matched comparators; mean age was 56 years, and 62% were women. The prevalence of various comorbidities was higher among patients in the PND group, including digestive disorders (31% vs. 17% for comparison group), circulatory disorders (29% vs. 22%), and depression (4% vs. 3%) (all *p *< 0.01). Receipt of prescriptions for pain-related pharmacotherapy also was higher among PND patients, including nonsteroidal anti-inflammatory drugs (56% of PND patients had one or more such prescriptions vs. only 22% in the comparison group), opioids (49% vs. 12%), tricyclic antidepressants (20% vs. 1%), and antiepileptics (12% vs. 1%) (all *p *< 0.01). PND patients also averaged significantly more GP visits (22.8 vs. 14.2) and referrals to specialists (2.8 vs. 1.4) over one year (both comparisons *p *< 0.01).

**Conclusions:**

Patients with PNDs under the care of GPs in the UK have relatively high levels of use of healthcare services and pain-related pharmacotherapy.

## Background

Neuropathic pain results from dysfunction of either the peripheral nerves or, less commonly, the central nervous system [1,2]. Neuropathic pain can be difficult to treat, and often requires the use of antiepileptic drugs (AEDs) and/or tricyclic antidepressants (TCAs) instead of--or in addition to--agents that are often used to treat nociceptive pain, such as nonsteroidal anti-inflammatory drugs (NSAIDs) and opioids. Previous guidelines for the treatment of painful neuropathies recommended a stepwise approach to treatment, with TCAs and/or AEDs used initially, followed by other agents (e.g., duloxetine, opioids) as needed. Pain clinics and/or psychological support also were recommended for patients whose pain remained inadequately controlled following multiple trials with different drugs [3,4]. Currently, TCAs, selected AEDs (i.e., gabapentin, pregabalin, carbamazepine), serotonin-norepinephrine reuptake inhibitor (SNRI) antidepressants (i.e., duloxetine, venlafaxine), and topical lidocaine are all recommended as first- and/or second-line therapy for pharmacologic management of painful neuropathies; tramadol and opioids are now recommended as second- and/or third-line therapy [5,6].

In contrast to the growing body of literature on the etiology, pathophysiology, and treatment of neuropathic pain, relatively little has been reported about the clinical characteristics and costs of patients with painful neuropathic disorders (PNDs) in clinical practice, including their levels of use of pain-related pharmacotherapy and healthcare services. These issues were examined by the authors in two prior studies in which patients with PNDs were compared with an equal number of age-and sex-matched comparators [7,8]. In the first study, 55,686 patients with PNDs in the US were identified during calendar-year (CY) 2000. In the second study, 275,685 patients with PNDs who were seen by general practitioners (GPs) in Germany between 1 August 2005 and 31 July 2006 were identified. In both studies, the prevalence of various comorbidities--including fibromyalgia, osteoarthritis, and depression--was much higher in patients with PNDs, as was the use of a range of pain-related medications, including opioids, AEDs, antidepressants, and benzodiazepines. Patients with PNDs received NSAIDs and opioids to a much greater extent than one would expect if treatment guidelines relevant for the time period of either study had been followed. Lachaine and colleagues used similar methods to examine the burden of PNDs among patients in Quebec [9]. Similar to findings reported for patients in the US and Germany, patients with PNDs (n = 4912) were reported to be more likely than their age- and gender-matched comparators to have comorbidities; they also had higher levels of use of pain-related medications, such as AEDs, antidepressants, opioids, and NSAIDs [9].

There is some evidence that patients with PNDs in the UK are similarly treated. In their study of approximately 25,000 patients with post-herpetic neuralgia, trigeminal neuralgia, phantom limb pain, or painful diabetic neuropathy newly diagnosed by a GP in the UK between CY1992 and CY2002, Hall et al. reported that the medications most commonly prescribed were amitriptyline, carbamazepine, coproxamol, codydramadol, and codeine + paracetamol [10]. In a second study that also focused on patients with these four PNDs in a UK GP database between May 2002 and July 2005, Hall et al. found that the most commonly prescribed drugs included TCAs, AEDs, and opioids [11]. A study by Gore and colleagues identified 30,999 patients with PNDs in a UK GP database in CY2001 (16,690 with pain predominantly neuropathic in nature ["pure"], and 14,309 with pain likely to have both nociceptive and neuropathic components ["mixed"]) [12]. Use of medications with proven efficacy in neuropathic pain (e.g., AEDs, TCAs) was reported to be low, although levels of use were higher in patients with "pure" versus "mixed" neuropathic pain. Many PND patients also had been prescribed agents that have not typically demonstrated efficacy in neuropathic pain (e.g., NSAIDs).

While providing important insights into the treatment of PNDs in the UK, these studies are not without their limitations. The two studies by Hall and colleagues were limited to the four above-noted PNDs; the degree to which their findings are generalizeable to the overall population of patients with PNDs is unknown. Moreover, none of these studies compared healthcare utilization and pharmacotherapy between patients with PNDs versus those without these disorders. We address this issue in the study described below, focusing attention on patients under the care of GPs in the UK.

## Methods

Data were obtained from The Health Improvement Network (THIN) database, which consists of patient-level information on GP encounters from approximately 300 computerized GP practices throughout the UK. All practices registered with THIN use a clinical management system provided by In Practice Systems Ltd. (INPS). Many practices have contributed over 15 years of data to THIN, and more than 5 million patients are represented in the database. The database documents all patient care by GPs, and includes extensive information on diagnoses and treatments; it is designed to be representative of GP practices throughout the UK. All patient identifiers in the database are fully encrypted to protect patient confidentiality.

Available information in the THIN database includes date of service, diagnoses (in READ format), actions taken (e.g., referrals to other providers [i.e., specialists]), and medications prescribed--including the prescribing date and the quantity prescribed. Selected demographic information is also available, including patient age and gender. All patient-level data can be arrayed chronologically to provide a detailed, longitudinal profile of all medical and pharmacy services rendered by participating GPs.

The study protocol was submitted for review to--and subsequently approved by--the UK National Health Service (NHS) Multi-Centre Research Ethics Committee (MREC).

The study sample consisted of all patients, aged ≥ 18 years, with one or more visits to GPs between 1 January 2006 and 31 December 2006 ("study period") (the most recent one-year period for which data were available at the time of study) at which a diagnosis of PND was noted (Appendix). A comparison group also was constituted, consisting of patients who did not have any GP encounters with noted diagnoses of PNDs during the same period; they were randomly selected and matched to PND patients based on age (within 1 year) and sex. (Patients in the comparison group could have other painful disorders, however, such as osteoarthritis and fibromyalgia.) All GP encounters were compiled for PND patients and patients in the comparison group over the 12-month study period.

The prevalence of selected clinically recognized comorbidities (e.g., arthritis, diseases of the circulatory system) was examined in both groups. Patients were deemed to have any of the conditions of interest if they had *any *encounters during the study period with a corresponding diagnosis code, or a prescription for a drug specific to that condition (e.g., diabetes was defined based on presence of corresponding [READ] diagnosis codes or prescriptions for any antidiabetic drugs).

The number of patients receiving prescriptions for pain-related and non-pain-related medications during the study period was examined. Medications were designated as "painrelated" based on their classification as analgesics or adjuvant medications in the World Health Organization's (WHO) "analgesic ladder" [13]. Although the WHO ladder was developed originally for cancer pain, the spectrum of pain-related medications also is used for the treatment of neuropathic pain [7,8,14-34]. Pain-related medications were defined to include: (1) AEDs; (2) benzodiazepines; (3) corticosteroids; (4) cyclo-oxygenase (COX)-2 inhibitors and other prescription NSAIDs; (5) muscle relaxants; (6) sedatives/hypnotics; (7) opioids (both short- and long-acting); (8) antidepressants (including TCAs, monoamine oxidase [MAO] inhibitors, and selective serotonin reuptake inhibitors [SSRIs]); (9) antimigraine agents; and (10) miscellaneous agents (injectable agents [e.g., bupivacaine], topical analgesics [e.g., lidocaine]). All other medications were designated non-pain-related.

Use of healthcare services during the 12-month study period was examined in terms of the numbers of GP visits, referrals from GPs to other healthcare providers (e.g., specialists), and hospitalizations; "sick notes" (physician-excused absences from work) also was examined. We did not attempt to attribute care specifically to the treatment of PNDs, because of inherent difficulties in attribution using electronic healthcare databases. We also did not attempt to examine nonpharmacologic treatments for PNDs, such as spinal cord stimulation and psychological support.

The statistical significance of differences in continuous measures between the PND and comparison groups was ascertained using paired t-tests or Wilcoxon signed-rank tests, as appropriate. McNemar's and Bowker's tests, as appropriate, were used to ascertain the statistical significance of differences in categorical measures. All analyses were conducted using PC-SAS^® ^v.8.4 [35].

## Results

We identified a total of 31,688 patients with one or more encounters at which PNDs were recorded between 1 December 2006 and 31 January 2006; a comparison group of the same size also was constituted, matched on age and gender. The most frequently noted PNDs were neuropathic back pain (46.4% of all PND patients), nerve impingement syndromes (e.g., carpal tunnel syndrome) (14.1%), and unspecified neuropathic pain (e.g., "neuritis, unspecified", "neuralgia unspecified") (9.7%) (Table 1).

**Table 1:** Distribution of painful neuropathic disorders*

Painful neuropathic disorder	Patients with painful neuropathies (N = 31,688)
Diabetic neuropathy	658 (2.1)

Diabetic neuropathy	812 (2.6)

Back pain with neuropathic involvement	14,689 (46.4)

Neck pain with neuropathic involvement	589 (1.9)

Cancer with neuropathic pain	0 (0.0)

Causalgia	73 (0.2)

Phantom limb pain	76 (0.2)

Trigeminal neuralgia	965 (3.0)

Atypical facial pain	702 (2.2)

Neuropathic pain, unspecified	

Neuralgia unspecified	1,737 (5.5)

Neuralgia, neuritis and radiculitis unspecified	2 (0.0)

Neuralgia, neuritis or radiculitis NOS	3 (0.0)

Neuritis unspecified	70 (0.2)

Neuropathic pain	698 (2.2)

Peripheral neuropathy	573 (1.8)

Polyneuropathy unspecified	2 (0.0)

Any of above	3,085 (9.7)

Nerve impingement syndromes	

Carpal tunnel syndrome	548 (1.7)

CTS-Carpal tunnel syndrome	3,379 (10.7)

Cubital tunnel syndrome	20 (0.1)

Median nerve compression in forearm	1 (0.0)

Meralgia paraesthetica	302 (1.0)

Morton's metatarsalgia	98 (0.3)

Morton's neuralgia	74 (0.2)

Nerve root and plexus compressions in	22 (0.1)

diseases EC	

Tarsal tunnel syndrome	9 (0.0)

Any of above	4,453 (14.1)

Other	

Acute infective polyneuritis	0 (0.0)

Acroparaesthesia-Schultze's type	0 (0.0)

Acroparaesthesia - unspecified	0 (0.0)

Alcoholic polyneuropathy	4 (0.0)

Burning feet syndrome	31 (0.1)

C/O paraesthesia	1,999 (6.3)

Hereditary and idiopathic peripheral neuropathy	2 (0.0)

Hereditary or idiopathic peripheral neuropathy NOS	11 (0.0)

Hereditary peripheral neuropathy	2 (0.0)

Hereditary sensory neuropathy	2 (0.0)

Inflammatory and toxic neuropathy	6 (0.0)

Korsakov's alcoholic psychosis with peripheral neuritis	0 (0.0)

Median nerve neuritis	2 (0.0)

Mononeuritis lower limb	7 (0.0)

Mononeuritis multiplex	3 (0.0)

Mononeuritis of unspecified site NOS	1 (0.0)

Mononeuritis of upper limb and mononeuritis multiplex	1 (0.0)

Mononeuritis upper limb NOS	2 (0.0)

Nerve root and plexus disorders	41 (0.1)

Nerve root or plexus disorder NOS	8 (0.0)

Neuralgic amyotrophy	6 (0.0)

Neuritis ulnar nerve	9 (0.0)

Neuropathy in association with hereditary ataxia	0 (0.0)

O/E-hyperaesthesia present	20 (0.1)

O/E-paraesthesia in hands	25 (0.1)

O/E-paraesthesia present	2 (0.0)

Other idiopathic peripheral neuropathy	2 (0.0)

Other idiopathic peripheral neuropathy NOS	0 (0.0)

Other mononeuritis lower limb	0 (0.0)

Other nerve root or plexus disorder	2 (0.0)

Other toxic agent polyneuropathy	0 (0.0)

Other toxic or inflammatory neuropathy	0 (0.0)

Other upper limb mononeuritis	1 (0.0)

Paraesthesia	0 (0.0)

Parsonage-Aldren-Turner syndrome	0 (0.0)

Peripheral neuritis in pregnancy	0 (0.0)

Peripheral neuropathy-hereditary or idiopathic	145 (0.5)

Policeman's disease	2 (0.0)

Polyneuropathy	23 (0.1)

Polyneuropathy due to drugs	0 (0.0)

Polyneuropathy in disease NOS	1 (0.0)

Polyneuropathy in disseminated lupus erythematosus	0 (0.0)

Polyneuropathy in porphyria	0 (0.0)

Polyneuropathy in rheumatoid arthritis	0 (0.0)

Polyneuropathy in sarcoidosis	0 (0.0)

Polyneuropathy in uraemia	0 (0.0)

Polyneuropathy in vitamin B deficiency	0 (0.0)

Radiculitis unspecified	7 (0.0)

Ramsey-Hunt syndrome	18 (0.1)

Toxic or inflammatory neuropathy NOS	0 (0.0)

Ulnar neuritis	139 (0.4)

Unspecified mononeuritis lower limb	0 (0.0)

D paraesthesia	1,756 (5.5)

X Polyneuropathy, unspecified	1 (0.0)

X Inflammatory polyneuropathy, unspecified	0 (0.0)

Any of above	4,281 (13.5)

Any of above	31,688 (100.0)

*All values are number of patients (%).

Mean (SD) age of patients in both groups was 56.1 (16.6) years, and 61.6% were women (Table 2). Nearly one-third of patients (32.9%) were ≥ 65 years of age. The prevalence of various comorbidities was higher among PND patients, including diseases of the respiratory system (33.1% vs. 24.6%, respectively), diseases of the digestive system (31.5% vs. 17.3%), diseases of the circulatory system (28.6% vs. 21.6%), depression (4.3% vs. 2.6%), and anxiety (2.8% vs. 1.7%) (all *p *< 0.01). Approximately one in five PND patients (i.e., 23%) had at least one encounter during the study period with a diagnosis of "symptoms, signs, and ill-defined conditions", compared with only 11.1% of patients in the comparison group (*p *< 0.01). Eighty-eight percent of patients with PNDs had two or more comorbidities noted by their GPs during the study period, versus 56.9% for the comparison group.

**Table 2:** Demographic and clinical characteristics of study subjects*

	Patients with painful neuropathies (N = 31,688)	Comparison group (N = 31,688)	*P*-Value
**Characteristic**			---

Age group, years			

18-44	8,621 (27.2)	8,621 (27.2)	

45-54	6,056 (19.1)	6,056 (19.1)	

55-64	6,577 (20.8)	6,577 (20.8)	

≥ 65	10,434 (32.9)	10,434 (32.9)	

Mean (SD)	56.1 (16.6)	56.1 (16.6)	---

Sex			---

Males	12,178 (38.4)	12,178 (38.4)	

Females	19,510 (61.6)	19,510 (61.6)	

Comorbidities			

Infectious and parasitic diseases	5,822 (18.4)	3,308 (10.4)	<0.01

Neoplasms	1,787 (5.6)	1,281 (4.0)	<0.01

Any endocrine, nutritional and metabolic diseases, and immunity disorders	10,699 (33.8)	7,604 (24.0)	<0.01

Diseases of the blood and blood-forming organs	2,487 (7.8)	1,507 (4.8)	<0.01

Any anxiety	897 (2.8)	547 (1.7)	<0.01

Any depression	1,365 (4.3)	827 (2.6)	<0.01

Any disease of the nervous system and sense organs	14,691 (46.4)	5,854 (18.5)	<0.01

Any disease of the circulatory system	9,071 (28.6)	6,837 (21.6)	<0.01

Any disease of the respiratory system	10,485 (33.1)	7,786 (24.6)	<0.01

Any disease of the digestive system	9,970 (31.5)	5,488 (17.3)	<0.01

Any disease of the genitourinary system	5,019 (15.8)	3,497 (11.0)	<0.01

Complications of pregnancy, childbirth, and the puerperium	325 (1.0)	241 (0.8)	<0.01

Any disease of the skin and subcutaneous tissue	9,773 (30.8)	7,136 (22.5)	<0.01

Diseases of the musculoskeletal system and connective tissue			

Back pain	15,444 (48.7)	693 (2.2)	<0.01

Cervical pain	2,011 (6.3)	615 (1.9)	<0.01

Arthritis	2,116 (6.7)	1,203 (3.8)	<0.01

Fibromyalgia	76 (0.2)	11 (0.0)	<0.01

Other body/joint pain	10,481 (33.1)	3,832 (12.1)	<0.01

Other	1,993 (6.3)	917 (2.9)	<0.01

Any disease of the musculoskeletal system and connective tissue	23,529 (74.3)	6,233 (19.7)	<0.01

Congenital anomalies	81 (0.3)	44 (0.1)	<0.01

Symptoms, signs, and ill- defined conditions			

Fatigue	16 (0.1)	12 (0.0)	0.45

Headache	392 (1.2)	79 (0.2)	<0.01

Chest pain	387 (1.2)	216 (0.7)	<0.01

Abdominal pain	640 (2.0)	351 (1.1)	<0.01

Anxiety-related symptoms	0 (0.0)	0 (0.0)	---

Gastric-related symptoms	3 (0.0)	1 (0.0)	0.32

Other	6,420 (20.3)	2,989 (9.4)	<0.01

Any symptoms, signs, and ill-	7,320 (23.1)	3,507 (11.1)	<0.01

defined conditions			

Any injury and poisoning	2,991 (9.4)	2,021 (6.4)	<0.01

Any sleep disorder	528 (1.7)	257 (0.8)	<0.01

Any of above**	31,447 (99.2)	24,768 (78.2)	<0.01

Number of comorbidities**			<0.01

0	241 (0.8)	6,920 (21.8)	

1	3,632 (11.5)	6,757 (21.3)	

2	4,617 (14.6)	5,648 (17.8)	

3	4,883 (15.4)	4,222 (13.3)	

≥ 4	18,315 (57.8)	8,141 (25.7)	

*Unless otherwise indicated, all values are number (%).

**Excluding painful neuropathic disorders.

The number of patients who received prescriptions from their GPs for pain-related pharmacotherapy was approximately twice as high in the PND group than in the comparison group (82.9% vs. 43.4%,, respectively; *p *< 0.01), including "traditional" analgesics, such as NSAIDs and opioids, as well as "adjuvant" medications, such as AEDs, benzodiazepine, and tricyclic antidepressants (Table 3). The pain-related medications most commonly prescribed by GPs to patients with PNDs included NSAIDs (including COX-2 inhibitors) (56.5% of PND patients vs. 22.3% in the comparison group), opioids (49.5% vs. 13.8%), antidepressants (primarily TCAs) (29.9% vs. 9.6%), benzodiazepines (13.0% vs. 4.9%), and AEDs (12.1% vs. 0.9%) (all *p *< 0.01).

**Table 3:** Use of pain-related medications among study subjects*

	Patients with painful neuropathies (N = 31,688)	Comparison group (N = 31,688)	*P*-Value
Number receiving			

Antiepileptic drugs	3,838 (12.1)	283 (0.9)	<0.01

Benzodiazepines	4,127 (13.0)	1,546 (4.9)	<0.01

Corticosteroids	3,297 (10.4)	1,563 (4.9)	<0.01

Nonsteroidal anti- inflammatory drugs			

Cyclooxygenase-2 inhibitors	730 (2.3)	209 (0.7)	<0.01

Other nonsteroidal anti- inflammatory drugs	17,597 (55.5)	6,946 (21.9)	<0.01

Any of above	17,902 (56.5)	7,059 (22.3)	<0.01

Muscle relaxants	422 (1.3)	70 (0.2)	<0.01

Sedatives/hypnotics	1,548 (4.9)	749 (2.4)	<0.01

Opioids			

Short-acting opioids	15,587 (49.2)	4,369 (13.8)	<0.01

Long-acting opioids	719 (2.3)	66 (0.2)	<0.01

Any of above	15,674 (49.5)	4,388 (13.8)	<0.01

Antidepressants			

Tricyclic antidepressants	6,320 (19.9)	477 (1.5)	<0.01

Monoamine oxidase inhibitors	12 (0.0)	7 (0.0)	0.25

Selective serotonin reuptake inhibitors	3,397 (10.7)	2,259 (7.1)	<0.01

Other antidepressants	999 (3.2)	523 (1.7)	<0.01

Any of above	9,463 (29.9)	3,038 (9.6)	<0.01

Antimigraine			

Triptans	480 (1.5)	268 (0.8)	<0.01

Other antimigraine	231 (0.7)	110 (0.3)	<0.01

Any of above	647 (2.0)	351 (1.1)	<0.01

Miscellaneous			

Injectable analgesics	759 (2.4)	410 (1.3)	<0.01

Topical analgesics	4,258 (13.4)	2,502 (7.9)	<0.01

Other	107 (0.3)	49 (0.2)	<0.01

Any of above	4,941 (15.6)	2,896 (9.1)	<0.01

Any of above	26,265 (82.9)	13,745 (43.4)	<0.01

Number receiving			<0.01

None of the above	5,434 (17.1)	17,952 (56.7)	

1 of the above	7,877 (24.9)	8,047 (25.4)	

2 of the above	7,919 (25.0)	3,677 (11.6)	

3 of the above	5,264 (16.6)	1,368 (4.3)	

≥ 4 of above	5,194 (16.4)	644 (2.0)	

Any of above	26,265 (82.9)	13,745 (43.4)	<0.01

*Unless otherwise indicated, all values are number (%).

The number of patients receiving prescriptions for non-pain-related medications also was much higher in the PND group than in the comparison group, including general systemic anti-infectives (45.6% vs. 34.6%, respectively), ACE inhibitors (26.6% vs. 16.8%), antiulcerants (e.g., acid pump inhibitors, H2 blockers) (23.7% vs. 12.1%), dermatologicals (23.6% vs. 17.1%), and cough and cold preparations (21.1% vs. 16.4%) (all *p *< 0.01) (Table 4). Overall use of non-pain-related medications was substantially higher among PND patients versus patients in the comparison group--for example, 54.4% of the former group received prescriptions for three or more different types of non-pain-related medications during the study period, versus only 39.1% of the latter group.

**Table 4:** Use of selected non-pain-related medications among study subjects*

	Patients with painful neuropathies (N = 31,688)	Comparison group (N = 31,688)	*P*-Value
Number receiving			

General anti-infectives systemic	14,451 (45.6)	10,953 (34.6)	<0.01

Cough and cold preparations	6,682 (21.1)	5,188 (16.4)	<0.01

Dermatologicals	7,464 (23.6)	5,415 (17.1)	<0.01

ACE inhibitors	8,421 (26.6)	5,327 (16.8)	<0.01

Antiulcerants	7,525 (23.7)	3,819 (12.1)	<0.01

Beta-blocking agents	6,148 (19.4)	4,446 (14.0)	<0.01

Anti-asthma and COPD products	5,534 (17.5)	3,450 (10.9)	<0.01

Drugs used in diabetes	4,553 (14.4)	3,996 (12.6)	<0.01

Diuretics	5,081 (16.0)	4,224 (13.3)	<0.01

Plain antispasmodics and anticholinergics	6,267 (19.8)	4,133 (13.0)	<0.01

Thyroid therapy	3,909 (12.3)	3,494 (11.0)	<0.01

Calcium antagonists	4,340 (13.7)	3,565 (11.3)	<0.01

Statins	4,377 (13.8)	1,900 (6.0)	<0.01

Antidiarrhoeals, oral electrolyte replacers and intestinal anti-inflammatories	3,336 (10.5)	2,116 (6.7)	<0.01

Non-narcotics and anti-pyretics	2,711 (8.6)	1,802 (5.7)	<0.01

Respiratory system and nasal preparations	2,558 (8.1)	1,722 (5.4)	<0.01

Angiotensin-II antagonists	2,532 (8.0)	1,764 (5.6)	<0.01

Heparins	2,412 (7.6)	1,573 (5.0)	<0.01

Ophthalmologicals	2,158 (6.8)	1,581 (5.0)	<0.01

Urologicals	2,614 (8.2)	1,406 (4.4)	<0.01

Systemic antihistamines	2,313 (7.3)	1,797 (5.7)	<0.01

Anti-gout preparations	2,305 (7.3)	1,531 (4.8)	<0.01

Nitrites and nitrates	2,016 (6.4)	991 (3.1)	<0.01

Mineral supplements	2,173 (6.9)	1,393 (4.4)	<0.01

Diagnostic agents	2,201 (6.9)	1,090 (3.4)	<0.01

Number receiving			<0.01

None of the above	5,114 (16.1)	8,329 (26.3)	

1 of the above	5,020 (15.8)	6,513 (20.6)	

2 of the above	4,329 (13.7)	4,460 (14.1)	

3 of the above	3,612 (11.4)	3,415 (10.8)	

≥ 4 of above	13,613 (43.0)	8,971 (28.3)	

Any of above	26,574 (83.9)	23,359 (73.7)	<0.01

*Unless otherwise indicated, all values are number (%)

ACE: Angiotensin-converting enzyme; COPD: Chronic obstructive pulmonary disease

Patients with PNDs averaged significantly more GP visits during the 12-month study (mean [95% CI] = 22.8 [22.6, 23.0] vs. 14.2 [14.1, 14.3] for comparison group; *p *< 0.01) (Table 5). They also had approximately twice as many referrals to specialists (2.8 [2.8, 2.9] vs. 1.4 [1.4, 1.4]) and hospitalizations (0.2 [0.2, 0.2] vs. 0.1 [0.1, 0.1]) (both *p *< 0.01). A total of 16.5% of PND patients had one or more "sick notes" (i.e., physician-excused work absences) during the year, versus 7.0%, of the comparison group.

**Table 5:** Numbers of office visits, referrals, hospitalizations, and sick notes among study subjects*

	Patients with painful neuropathies (N = 31,688)	Comparison group (N = 31,688)	*P*-Value
Number of office visits			<0.01

0-5	3,314 (10.5)	9,680 (30.5)	

6-10	4,489 (14.2)	5,892 (18.6)	

11-15	4,602 (14.5)	4,641 (14.6)	

16-20	4,295 (13.6)	3,696 (11.7)	

> 20	14,988 (47.3)	7,779 (24.5)	

Mean (95% CI)	22.8 (22.6-23.0)	14.2 (14.1-14.3)	<0.01

Median (IQR)	19 (11)	11 (4)	

Minimum	1	1	

Maximum	356	362	

Number of referrals			<0.01

0	11,136 (35.1)	17,875 (56.4)	

1	5,751 (18.1)	5,256 (16.6)	

2	3,664 (11.6)	2,641 (8.3)	

≥ 3	11,137 (35.1)	5,916 (18.7)	

Mean (95% CI)	2.8 (2.8-2.9)	1.4 (1.4-1.4)	<0.01

Median (IQR)	1.0 (0.0-4.0)	0.0 (0.0-2.0)	

Minimum	0	0	

Maximum	55	65	

Number of hospitalizations			<0.01

0	28,538 (90.1)	29,971 (94.6)	

1	2,124 (6.7)	1,175 (3.7)	

2	635 (2.0)	352 (1.1)	

≥ 3	391 (1.2)	190 (0.6)	

Mean (95% CI)	0.2 (0.2-0.2)	0.1 (0.1-0.1)	<0.01

Median (IQR)	0.0 (0.0-0.0)	0.0 (0.0-0.0)	

Minimum	0	0	

Maximum	23	38	

Number of sick notes			<0.01

0	26,453 (83.5)	29,474 (93.0)	

1	2,046 (6.5)	1,111 (3.5)	

2	1,109 (3.5)	505 (1.6)	

≥ 3	2,080 (6.6)	598 (1.9)	

Mean (95% CI)	0.5 (0.4-0.5)	0.2 (0.1-0.2)	<0.01

Median (IQR)	0.0 (0.0-0.0)	0.0 (0.0-0.0)	

Minimum	0	0	

Maximum	19	19	

*Unless otherwise indicated, all values are number (%).

*CI *confidence interval; *IQR *interquartile range

## Discussion

Over a 12-month period, patients with PNDs were approximately twice as likely to have been prescribed pain-related medications by their GPs than patients without evidence of these disorders (83% vs. 43%, respectively)-most commonly, NSAIDs and opioids. Despite the proven efficacy of antidepressants (especially, TCAs) in treating neuropathic pain, which has led to recommendations regarding their use as first-line therapy [4-6,36,37], only 30% of PND patients received prescriptions for these agents, and only 12% had been prescribed AEDs. These patterns of medication prescribing may indicate that UK GPs may be more comfortable with "traditional" analgesics for the treatment of neuropathic pain, which may have been appropriate to some degree, given that evidence of efficacy of opioids in neuropathic pain was available during the study period [37-41]. (We also note that current treatment recommendations, which were promulgated after the end of our study period, list opioids as second- and/or third-line options for neuropathic pain [5,6].) On the other hand, we were unable to ascertain the number of patients who received TCAs and AEDs in the past and discontinued these medications due to adverse effects or lack of efficacy. We also note that back pain with neuropathic involvement-the most frequently noted PND (~46% of all PND patients)-is often associated with both neuropathic and nociceptive pain; the latter often responds well to NSAIDs and/or opioids.

Patients with PNDs also had more GP encounters with diagnoses of other medical problems (e.g., digestive, circulatory, and respiratory disorders, depression, anxiety) compared with their age-and sex-matched peers without evidence of PND; they also had higher levels of use of non-pain-related medications, such as anti-ulcerants, systemic anti-infectives, and dermatologicals. Patients with PNDs averaged 10 additional GP office visits over 1 year, and twice as many specialist referrals and hospitalizations; 17% had at least one physician-excused absence from work versus as opposed to only 7.0% in the comparison group. Other studies of US and German patients have reported similar findings [7,8].

While the precise reasons for these associations are unclear, a number of possible explanations come to mind. For one, some comorbid conditions may be etiologically related to PNDs-for example, diabetes, vascular complications thereof, and diabetic peripheral neuropathy-and such associations therefore are not unexpected. More generally, since neuropathic disorders are often chronic and difficult to treat, patients with PNDs may present more frequently to their GPs than patients without these disorders. More frequent visits may lead to more opportunistic case findings, and hence a higher rate of clinical recognition of many complaints and diseases that otherwise might have gone undiagnosed and/or untreated. Difficulties in diagnosing neuropathic disorders also may have yielded spuriously high rates of comorbid conditions among PND patients. On the other hand, since THIN does not have a direct link to hospital data and/or sick notes, our findings may actually underestimate the prevalence of various comorbidities. Further study is needed to better understand how common these comorbidities are in patients with PNDs, as well as the relationship between these conditions and PNDs.

In their study utilizing another electronic database containing information from GP encounters in the UK, Gore and colleagues reported that NSAIDs were the most commonly dispensed medication among patients with "pure" PNDs (i.e., conditions in which pain was believed to be predominantly neuropathic in nature) (53% of all identified patients with PNDs) [12]; 17% received TCAs, 12% received AEDs, 11% received a second-generation antidepressants (e.g., SSRIs), and 9% received opioids. We generally observed much higher levels of prescribing of these medications in our study, the only exception being NSAIDs. Of particular note, the number of PND patients who were prescribed opioids was almost fivefold higher in our study than that reported by Gore and colleagues. While the precise reasons for this difference are unclear, it may be due to differences in operational definitions of study measures (e.g., how combination products containing NSAIDs and opioids were classified) and/or differences in the study databases. Another possible reason may be changes in practice and prescribing patterns that have occurred between the timeframes of the Gore et al. study (CY2001) and ours (CY2006), although the magnitude of difference seems too high to be explained by this factor alone.

Patterns of prescribing of pain-related pharmacotherapy among our study subjects also differed substantially from those reported by Hall and colleagues, who used the same database to examine patterns of pharmacotherapy among patients with post-herpetic neuralgia (N = 1923), trigeminal neuralgia (N = 1862), phantom limb pain (N = 57), and painful diabetic neuropathy (N = 1444) between May 2002 and July 2005 [11]. Specifically, Hall et al. reported substantially higher percentages of patients receiving prescriptions for AEDs (32.5% vs. 12.1% in our current study) and TCAs (41.3% vs. 19.9%); we observed higher percentages of patients receiving prescriptions for opioids (49.5% vs. 29.1% in Hall et al.), NSAIDs (56.5% vs. 30.6% ["non-opioid analgesics"]), and other antidepressants (10.0% vs. 3.7%). The biggest reason for this discrepancy is likely the sample selected-namely, Hall et al. focused on only four PNDs, while we included several other types of PNDs (e.g., back pain with neuropathic involvement, causalgia, nerve impingement syndromes).

Our study has several limitations. Perhaps most important, our findings are based on information from CY2006. Treatment guidelines changed after CY2006 and now recommend a number of different medications as first-line therapy for PNDs (e.g., TCAs, selected AEDs, selected SNRIs, topical lidocaine) [5,6]; the degree to which our findings represent current practice patterns in the UK is unknown.

Second, information on medication use was limited to drugs prescribed by GPs. Thus, to the extent that patients with PNDs (or those in the comparison group, for that matter) used medications available for direct retail purchase (i.e., without a prescription) and/or received "private prescriptions" (i.e., prescriptions wholly paid for by the patient [as opposed to the NHS), we may have underestimated total medication use.

Third, we do not know the reason(s) why particular medications were prescribed (e.g., for the treatment of PNDs or for pain associated with other conditions). Since some medications designated as "pain-related" also may be used to treat conditions that are not typically associated with pain (e.g., AEDs and seizure disorders, antidepressants and depression), it would be incorrect to infer that these agents were used exclusively for the treatment of neuropathic pain. However, only 4% of PND patients-and 3% of those in the comparison group-had any encounters at which a diagnosis of depression was recorded; corresponding values for epilepsy were 0.2% and 0.1%, respectively (data not shown). Similarly, given the widespread prevalence of comorbid pain-related conditions among patients with PNDs, as well as the possibility of "mixed" pain (i.e., neuropathic and nociceptive) among those with back pain with neuropathic involvement, it is possible that NSAIDs (and other pain-related medications for that matter) may have been prescribed for the treatment of pain that was not neuropathic in origin (e.g., triptans for migraine headache). Since we did not have access to medical records, the degree to which this actually occurred must remain conjectural.

Fourth, the study database is limited to information from GP encounters. Given the large numbers of PND patients with medical and psychiatric comorbidities-as well as the difficulty of treating PNDs-it is reasonable to expect that some patients in the study sample also had encounters with healthcare providers other than GPs.

Fifth, the GP-centric records of referrals, sick notes, and hospitalizations that are available in the study database are complete only to the extent that GPs actually record such information; to the extent that this information is not recorded by GPs, our findings may represent underestimates of healthcare utilization and prescribing patterns in this population.

Sixth, study subjects were selected based on evidence of at least one GP encounter during which a diagnosis of a PND was recorded during the one-year study period. Thus, patients who were exclusively under the care of specialists (e.g., neurologists, pain specialists) for their neuropathic pain, but who saw GPs for other reasons, would not necessarily have had GP encounters at which PNDs were noted. Not only would these patients not have been designated as having PNDs, but they also could have been selected for inclusion in the comparison group. On the other hand, the database does not contain information on how physicians rendered their diagnoses. Given that some types of PNDs are more difficult to diagnosis than others [42-44], and others are not exclusively neuropathic in origin (e.g., atypical facial pain) [45], it is possible that we may have misclassified some patients as having PNDs who in fact suffered from nociceptive pain. Unfortunately, since the database does not contain information that would shed light on these issues, the nature and extent of any misclassification that might have occurred is unknown.

## Conclusions

Patients with PNDs under the care of GPs in the UK have relatively high levels of use of healthcare services and pain-related (and other) pharmacotherapy. Because treatment guidelines evolved while our study was underway, it is unknown whether patterns of pharmacotherapy that we observed are representative of clinical practice in the UK today.

## Competing interests

Funding for this research was provided by Pfizer Inc., New York, NY. Two of the study authors--Dr. Sadosky, an employee of Pfizer Inc., and Dr. Dukes, an employee of Pfizer at the time this research was conducted--were involved with the design of the study, data analysis and interpretation, manuscript preparation, and publication decisions. Mr. Berger, Dr. Edelsberg, and Dr. Oster, who are employed by Policy Analysis Inc., were involved with the design of the study, data analysis and interpretation, manuscript preparation and publication decisions. Policy Analysis Inc. received funding to support manuscript development.

## Authors' contributions

All authors (AB, AS, ED, JE and GO) helped with all aspects of this study (i.e., conceptualization and design of study, analysis and interpretation of data, manuscript preparation and review). All authors read and approved the final manuscript.

## Disclosures

This research was funded by Pfizer Inc., New York, NY. Mr. Berger, Dr. Edelsberg, and Dr. Oster are employed by Policy Analysis Inc., an independent contract research organization. Dr. Dukes is a former employee of Pfizer Inc. Dr. Sadosky is currently employed by Pfizer Inc.

## Appendix

Table 6 sets forth all diagnoses (in READ format) used to identify patients with painful neuropathic disorders.

**Table 6:** Painful neuropathic disorders

	ICD-10 diagnosis codes
**Painful neuropathic disorders**	

"Pure" painful neuropathic disorders	

Diabetic neuropathy	250 AT, 250 F, C106.00, C106.11, C106.12, C106.13, C106000, C106100, C106y00, C106z00, C108200, C108211, C108212, C108B00, C108B11, C108B12, C108C00, C108C11, C108C12, C108J00, C108J11, C108J12, C109200, C109211, C109212, C109A00, C109A11, C109A12,C109B00, C109B11, C109B12, C10A400, C10E200, C10E211, C10E212, C10F200, C10F211, C10FH00, C10FH11, F345000, F35z000, F372.00, F372.11, F372.12, F372000, F372100, F381311, F3y0.00

Post-herpetic neuralgia	054 G, 355 PH, A531.11, A531200, A531300, A531500, A531511, F300.00, F374400

Back pain with neuropathic involvement	352, 7286, 3499E, 353 A, 353 C, 353 HP, 353 NP, 353 NT, 353 PP, 353 T, 355 AR, 355 AT, 7251D, 7285A, 7288LM, 7288RP, 7289B, 7289CE, F161400, F163.00, F163000, F163200, F163z00, F16y.00, F16y000, F16y100, F16yz00, F16z.00, F16z.11, F16z.12, F246.00, F246000, F246100, F246z00, F29y400, F29y411, F337100, F337200, F337200, F337300, F350.00, F378.00, N113.00, N113000, N113100, N113200, N115.00, N115000, N115100, N115200, N11B.00, N11B000, N11B100, N11B200, N11C.00, N11C000, N11C100, N11C200, N11y200, N11z100, N129.00, N129.11, N129000, N129200, N129300, N129z00, N12B.00, N12B100, N12B200, N12C400, N134.11, N142000, N143.00, N143.11, N144.00, N144.00, N144000, N144100, N144z00, N144z00, Nyu6200, Nyu6300, Nyu7300, Nyu7400

Neck pain with neuropathic involvement	7284, 3470 CE, 352 CS, 355 AS, 357 J, F330300, N111.00, N111000, N111100, N111200, N111300, N119.00, N119000, N119100, N119200, N129100, N12B000, N12zH00, N134.00, N134.12, N134.12

Cancer with neuropathic pain	F337000, F373.00, Fyu7400

Causalgia	355 CL, F344.00, N337.12, N337111

Phantom limb pain	7816PL, 7816PM, 7816PN, F336.00, F336000

Trigeminal neuralgia	1475.00, 7021400, 7023000, 7024400, 7024411, 7025400, 7027400, 7028400, 7028511, 351 A, 355 JW, 357 H, F301.00, F301000, F301z00, Fyu6000

Atypical facial pain	351 AF, 351 BA, F302.00, F321.00

Other painful neuropathies	1B46.00, 261 BR, 276 NR, 29B4.00, 29B5.00, 29B5000, 3479DT, 351 OC, 7871 HA, 7871 HB, 792 N, 792 PN, A531111, A72x100, C262300, C34y400, F335.00, F335.11, F342.11, F342000, F370100, F370z00, FyuAE00, G73y400, G73y411, G73y500, G73y511, G73y600, R020700, 340.12, 352, 355.00, 357, 7283, 7288, 3032R, 3039PN, 3499M, 352 L, 352 LN, 354 P, 355 A, 355 AB, 355 AQ, 355 AV, 355 B, 355 C, 355D, 357 C, 357 K, 357 KL, 357 KP, 357 LN, 357 ME, 357 NL, 357TT, 7283BR, 7284RP, 984 N, 9851N, 9909ML, 'E011100, F33..00, F337.00, F33y.00, F33z.00, F34..00, F340.00, F340.12, F341000, F341100, F345.00, F34y.00, F34z.00, F35..00, F351.00, F355.00, F356000, F356100, F356100, F35x.00, F35y.00, F35z.00, F35z.11, F36..00, F360.00, F360z00, F362.00, F364.00, F365.00, F366.00, F367.00, F36y.00, F36yz00, F36z.00, F37..00, F37..11, F370.00, F371.00, F371000, F371100, F371200, F371z00, F374.00, F374000, F374100, F374200, F374300, F374500, F374600, F374700, F374800, F374900, F374A00, F374z00, F375.00, F376.00, F377.00, F37x.00, F37y.00, F37y000, F37z.00, F37z.11, Fyu1300, Fyu6.00, Fyu6500, Fyu6A00, Fyu6B00, Fyu6C00, Fyu6D00, Fyu7.00, Fyu7100, Fyu7200, Fyu7300, Fyu7500, Fyu7600, Fyu7700, Fyu7800, Fyu7900, Fyu7B00, Fyu7C00, FyuAC00, L164.00, L164000, L164200, L164300, L164400, L164z00, N134.14, N1y0.00, N1y0.00, N242.00, N242000, N242100, N242200, N242300, N242z00, N242z11

## Pre-publication history

The pre-publication history for this paper can be accessed here:

http://www.biomedcentral.com/1471-2377/12/8/prepub
